# Risk factors for coronary artery disease in young patients with stable angina pectoris

**DOI:** 10.3906/sag-1905-56

**Published:** 2019-08-08

**Authors:** Mustafa DURAN, Deniz ELÇİK, Sani MURAT, Fatih ÖKSÜZ, İbrahim Etem ÇELİK

**Affiliations:** 1 Department of Cardiology, Ankara Research and Education Hospital, Ankara Turkey; 2 Department of Cardiology, Faculty of Medicine, Erciyes University, Kayseri Turkey

**Keywords:** Coronary artery disease, stable angina pectoris, young patients

## Abstract

**Background/aim:**

We aimed to investigate the relationship between risk factors and the presence of coronary artery disease (CAD) in a young population with stable angina pectoris (SAP).

**Materials and methods:**

A total of 571 individuals younger than 60 years old, admitted to the outpatient clinic with chest pain and referred for coronary angiography between January 2015 and December 2017, were included in the study. All clinical and biochemical parameters were documented in the hospital records. Coronary angiography of patients was monitored from records. The individuals were divided into two groups. The patient group consisted of 363 individuals with at least one-vessel stenosis of ≥70%, and the control group consisted of 208 individuals with normal coronary angiography. We compared the traditional and nontraditional risk factors of these two groups in terms of the presence of CAD.

**Results:**

Prevalence of male sex and smoking were higher in the patient group, and the prevalence of hypertension and diabetes were similar in the two groups. In the patient group, mean age, blood cholesterols, serum gamma-glutamyltransferase, hemoglobin, and white blood cell and lymphocyte levels were higher, while estimated glomerular filtration rate (eGFR), high-density lipoprotein cholesterol (HDL-C), platelets, and neutrophil/lymphocyte and platelet/lymphocyte ratios were lower. Low eGFR and HDL-C levels, older age, male sex, smoking, and high levels of low-density lipoprotein cholesterol and lymphocytes were independent risk factors for the presence of CAD in young patients.

**Conclusion:**

Contrary to studies performed in the elderly, traditional and nontraditional risk factors could not exactly predict the presence of CAD in a young population with SAP.

## 1. Introduction

Coronary artery disease (CAD) is a major cause of death worldwide [1]. It has commonly been associated with aging; however, the frequency of CAD in young individuals has been increasing in recent years [2,3]. There is a growing interest in the identification and treatment of risk factors for CAD in young patients in order to decrease the incidence of CAD through risk modification and increase the quality of life through treatment [4,5]. Although traditional and nontraditional CAD risk factors are well defined for elderly patients with acute coronary syndrome (ACS), they are not fully clarified in younger people with stable angina pectoris (SAP). 

In order to provide earlier and better diagnosis of CAD in young adults, novel risk factors and markers have been evaluated in the literature [6–8]. However, these studies included specific cohorts, such as ACS [6–9]. Therefore, we aimed to investigate the relationship between traditional and nontraditional risk factors and the presence of significant atherosclerosis in a young population with SAP.

## 2. Materials and methods 

Using the Ankara Education and Research Hospital’s database, we identified all patients with SAP of <60 years of age who underwent coronary angiography (CAG) and had a measurement of hematological and biochemical markers before CAG between January 2015 and December 2017. Stable angina pectoris was diagnosed by clinical evaluation and stress tests, often with an exercise stress test. We included all patients with SAP, with the exception of the following exclusion criteria. We excluded patients with eGFR of <30 mL/min/1.73 m2. We also excluded patients with prior CAD, such as a history of myocardial infarction, coronary revascularization, congestive heart failure, cerebrovascular events, and peripheral vascular disease. Diabetes, hypertension, and smoking were deﬁned as conditions where a history of these conditions was documented in hospital records, regardless of duration or need for antidiabetic or antihypertensive agents. An estimated glomerular filtration rate (eGFR) was obtained by applying the Modification of Diet in Renal Disease (MDRD) study formula [10]. 

The major risk factors for cardiovascular disease (CVD) can be classified into 2 broad categories: traditional and nontraditional [11]. Traditional risk factors, such as diabetes, hypertension, smoking, and high levels of serum cholesterol, can be identified as biological features of patients that predict a well-defined outcome of CAD and lie directly in the biological causal pathway. They are strongly associated with the presence of CAD and explain almost 90% of events [11]. Nontraditional risk factors, such as inflammatory markers and low eGFR, can be identified as biomarkers or parameters that are involved in developing CAD that may or may not be causal.

Left and right coronary angiography was performed in multiple projections by the Judkins or Stones technique. Each angiogram was interpreted and the degree of coronary artery diameter stenosis was estimated visually by two independent cardiologists. Significant atherosclerosis was described as ≥70% of stenosis in coronary arteries. The patient group consisted of 363 young patients referred for CAG and diagnosed with SAP, with stenosis of ≥70% in at least one vessel. The control group consisted of 208 healthy individuals who were referred for CAG and had a normal coronary angiography.

The ethics committee approved this study. All procedures performed in studies involving human participants were in accordance with the ethical standards of the institutional and/or national research committee and the 1964 Helsinki Declaration and its later amendments, or comparable ethical standards.

### 2.1. Statistical analysis

Analyses were performed using the SPSS 18.0 for Windows (IBM SPSS Statistics, Chicago, IL, USA). Baseline data were presented as a percentage for categorical variables and as mean or median for numerical data, according to distributions. Continuous variables were compared using Student’s t-test for normally distributed variables and the Mann–Whitney U test for nonnormally distributed variables. Differences in baseline characteristics between groups were compared using chi-square tests and Fisher’s exact tests for categorical data. The independent associations between risk factors and the presence of CAD were analyzed using univariate and multivariate analysis. Covariates of parameters that were important in univariate analysis were added to the multivariate analysis model. Statistical significance was P < 0.05 for differences between groups.

## 3. Results 

Overall, the mean age was 49 ± 8 years, and the prevalence of risk factors was as follows: hypertension 37.8% (216), diabetes 42.2% (241), smoking 34.2% (195), and male sex 60.6% (346). Median eGFR was 92 (34–190) mL/min/1.73 m2, and 39.1% (223) of individuals had mild to moderate renal dysfunction (eGFR 30–89 mL/min/1.73 m2). The prevalences of male sex and smoking were significantly higher in the patient group (P < 0.001 for both; Table 1). The prevalences of hypertension and diabetes were similar in the two groups (P > 0.005 for both; Table 1). The control group was younger than the patient group (46 ± 10 vs. 52 ± 6, P < 0.001). Levels of eGFR, high density lipoprotein cholesterol (HDL-C), and platelets were lower in the patient group than in the control (P < 0.05 for all; Table 1). Triglyceride, low-density lipoprotein cholesterol (LDL-C), total cholesterol, serum gamma-glutamyltransferase (GGT), hemoglobin, white blood cell (WBC) levels, and lymphocyte levels were higher in the patient group than in the control (P < 0.05; Table 1). The strong inflammatory markers of neutrophil/lymphocyte ratio (NLR) and platelet/lymphocyte ratio (PLR) were lower in the patient group than the control (P = 0.019 and P < 0.001, respectively). Patients with mild to moderate renal dysfunction (eGFR between 30–90 mL/min/1.73 m2) were more prevalent in the patient group (170 patients, 46.8%) than in the control (53 patients, 25.5%; P < 0.001). According to the univariate and multivariate logistic regression models, low eGFR and HDL-C levels, older age, male sex, smoking, and high levels of LDL-C and lymphocytes were independent risk factors for the presence of CAD in young patients (Table 2).

**Table 1:** Baseline characteristics of participants with and without coronary artery disease.

Variables	Patients group(n = 363)	Control group(n = 208)	P
Sex (male: n, %)	292 (80.4%)	54 (26.0%)	<0.001
Age (years)	52 ± 6	46 ± 10	<0.001
Diabetes (n, %)	143 (39.4%)	98 (47.1%)	0.072
Hypertension (n, %)	142 (39.1%)	74 (35.6%)	0.401
Smoking (n, %)	154 (42.4%)	41 (19.7)	<0.001
eGFR 30–89 (mL/min/1.73 m2)	170 (46.8%)	53 (25.5%)	<0.001
eGFR (mL/min/1.73 m2)	91 (34–190)	93 (43–196)	0.003
BUN (mg/dL)	31.7 ± 11.5	33.7 ± 7.9	0.057
Creatinine (mg/dL)	0.9 ± 0.3	0.8 ± 0.2	0.05
Triglyceride (mg/dL)	162 (35–956)	124 (41–514)	<0.001
HDL-C (mg/dL)	37 (10–72)	43 (17–87)	<0.001
LDL-C (mg/dL)	120 (37–296)	108 (31–290)	<0.001
Total cholesterol (mg/dL)	191 (63–393)	171 (33–281)	<0.001
Glucose (mg/dL)	110 (51–470)	97 (69–266)	0.025
BMI (kg/m2)	28.7 ± 4.5	31.0 ± 5.3	0.626
Uric acid (mg/dL)	5.6 ± 1.5	5.6 ± 1.6	0.804
Hemoglobin (mg/dL)	13.9 ± 1.7	13.1 ± 1.8	<0.001
White blood cell (×109/L)	8.8 ± 2.5	8.1 ± 2.4	0.001
Neutrophil (×109/L)	5.6 ± 2.3	5.4 ± 2.3	0.14
Lymphocytes (×109/L)	2.3 ± 0.8	2.0 ± 0.8	<0.001
NLR (%)	2.1 (0.5–21.1)	2.5 (0.73–19.6)	0.019
Platelet (×109/L)	234 (87–265)	248 (42–455)	0.041
PLR (%)	105 (28–376)	128 (32–435)	<0.001
Mean platelet volume (fL)	9.9 ± 1.3	10.1 ± 1.5	0.099
AST (U/L)	22 (4–224)	21 (11–150)	0.455
ALT (U/L)	21 (7–189)	19 (7–155)	0.196
GGT (U/L)	28 (10–164)	22 (11–155)	0.005
Total bilirubin (mg/dL)	0.5 (0.1–4.7)	0.6 (0.2–1.5)	0.123

eGFR: Estimated glomerular filtration rate; BUN: blood urea nitrogen; HDL-C: high-density lipoprotein cholesterol; LDL-C: low-density lipoprotein cholesterol; BMI: body mass index; NLR: neutrophil lymphocytes ratio; PLR: platelet lymphocytes ratio; AST: aspartate aminotransferase; ALT: alanine aminotransferase; GGT: gamma-glutamyltransferase.

**Table 2:** Univariate and multivariate analysis of predictors of coronary artery disease in young patients with stable angina pectoris.

Covariates	Univariate analysis			Multivariate analysis		
	HR	95% CI	P	HR	95% CI	P
eGFR (30–90 mL/min/1.73 m2)	2.576	1.172–3.744	<0.001	2.220	1.267–3.879	0.005
Sex (male)	11.7	7.829–17.57	<0.001	9.452	4.925–18.13	<0.001
Age (years)	1.089	1.046–1.134	<0.001	1.090	1.047–1.135	<0.001
Smoking (n, %)	3.001	2.012–4.478	<0.001	5.161	2.552–10.43	<0.001
TG (mg/dL)	1.007	1.004–1.009	<0.001	1.003	0.999–1.006	0.158
HDL-C (mg/dL)	0.944	0.927–0.961	<0.001	0.963	0.935–0.991	0.010
LDL-C (mg/dL)	1.008	1.003–1.013	<0.001	1.014	1.001–1.026	0.034
Total cholesterol (mg/dL)	1.011	1.007–1.015	<0.001	0.998	0.984–1.011	0.719
Glucose	1.007	1000–1013	0.054			
GGT (U/L)	1.019	0.996–1.042	0.106			
Hemoglobin	1.286	1.159–1.427	<0.001	0.888	0.746–1.056	0.179
White blood cell	1.122	1.039–1.212	0.003	0.945	0.843–1.060	0.334
Lymphocytes	1.522	1.206–1.920	<0.001	1.481	1.033–2.124	0.033
Platelet	0.995	0.995–1.000	0.003	1.000	0.996–1.004	0.926
Diabetes	0.730	0.517–1.029	0.073			
Hypertension	1.164	0.817–1.657	0.401			

TG: Triglyceride; HLD-C: high-density lipoprotein cholesterol; LDL-C: low-density lipoprotein cholesterol; GGT: gammaglutamyltransferase.

## 4. Discussion 

According to our results, hypertension and diabetes, two well-known traditional risk factors, could not predict CAD in young patients with SAP. Age, smoking, and lipid profiles could predict CAD in this population. This may be due to the age of our study participants; they had not been exposed to risk factors for as long as older populations. Additionally, well-known nontraditional risk factors such as NLR, PLR, WBC, hemoglobin, and platelets could not predict CAD in young patients with SAP. This may be due to the characteristics of our study population, which consisted of SAP patients, not ACS patients. 

Several studies used the age range of 40–60 years to investigate clinical characteristics and outcomes in young patients with CAD [6,8,12,13]. Therefore, we used an age cutoff of 60 for young patients in our study.

Traditional risk factors, such as hypertension, diabetes, smoking, and dyslipidemia, are present in most patients with CAD [6,10,14]. In our study, the number of male patients was significantly higher in the patient group than in the control, which was in accordance with this general information [15,16]. Another important traditional risk factor of CAD is older age [8]. Our study group consisted of patients ˂60 years of age, but when we compared the average age in each group, the mean age of the patient group was higher than that in the control group (52 vs. 46 years, P < 0.001). When we compared the two groups according to lipid profiles, total cholesterol and LDL-C and triglyceride levels were significantly higher and HDL-C levels were significantly lower in the patient group than in the control, in accordance with previous study results [17]. Smoking is the most important risk factor for CAD, with the contribution ranging from 62% to 90% according to various studies [9,17]. In accordance with this, the number of smoking patients was greater in the patient group than in the control. In our study, some conventional risk factors were not significantly different between the groups. For example, the frequencies of hypertension and diabetes were similar between the groups (P = 0.072 and P = 0.401). Obese patients manifest CAD at a younger age, and Lakka et al. reported that abdominal obesity is an independent risk factor for ACS in middle-aged men [18]. However, in our study, body mass index was not significantly different between groups. We did not evaluate abdominal obesity. 

Chronic kidney disease is a traditional risk factor, but mild to moderate renal dysfunction is not a traditional risk factor for CAD [19]. Several studies (the MATISS and ARIC trials) have shown that mild to moderate renal dysfunction is associated with increased risk of incident CVD [19,20]. Eisen et al. reported that lower eGFR is associated with higher cardiovascular events in men and women of all ages without prior CVD, particularly in the range of 100 mL/min/1.73 m2 < eGFR < 130 mL/min/1.73 m2 [21]. These results indicate that lower eGFR may be an independent risk marker for incident CVD. In our study, levels of eGFR were lower and the number of patients with mild to moderate renal dysfunction (eGFR 30–90 mL/min/1.73 m2) was higher in the patient group than in the control (P = 0.003 and P < 0.001, respectively).

Anemia is a risk factor for CVD and an independent predictor of adverse outcomes in ACS [22]. Inflammatory mediators may be triggered by anemia, and inflammation is associated with atherosclerotic progression [22]. In our study, hemoglobin was not significantly associated with CAD in young patients according to the multivariate analysis. 

Serum inflammatory markers [such as WBC count, lymphocytes, neutrophil, NLR, PLR, and uric acid (UA)] and platelets play a pivotal role in the pathogenesis of atherosclerosis [23]. Investigators reported in several studies that plasma markers of inflammation (increased WBC and neutrophil counts) were associated with a higher risk of CAD [7]. In addition, a low blood lymphocyte count indicates reduced immunity and has been associated with cardiovascular complications in patients with CAD [23,24]. In the present study, the WBC and lymphocyte counts were higher and platelet count was lower in the patient group than in the control (P = 0.001, P < 0.001, P = 0.041, respectively). The NLR reflects the inflammatory status, and the PLR is a prognostic marker that reflects both the aggregation and inflammation pathway statuses [25]. Higher levels of NLR and PLR in patients with ACS were associated with an increased risk of adverse cardiovascular events at follow-up [26]. Bressi et al. reported that high levels of NLR were associated with an increased risk of 5-year adverse clinical events, whereas no significant difference was observed across tertiles of PLR in patients with SAP undergoing PCI [23]. In the present study, current inflammatory markers NLR and PLR were significantly lower in the patient group than in the control (P = 0.019 and P < 0.001, respectively). This was a result that contradicted the previous results found thus far. This may be due to the nature of our patients; they underwent CAG with the diagnosis of SAP, and there was no acute inflammatory status in our study group. We speculate that the relationship between inflammatory markers and the presence or prognosis of CAD is only valid in acute cases, not in stable patients. We suggest that inflammation does not play an important role in the pathogenesis of atherosclerosis in stable patients. We have also obtained evidence supporting this proposition as follows: Lv et al. reported that serum UA was associated with the presence of CAD in nonsmokers ≤35 years of age [27]. Several studies reported an association between serum UA and atherosclerosis, but in some of these UA was not found to be a significant marker for atherosclerosis in multivariate analysis [28]. In our study, no statistically significant difference was found between patients with and without CAD, in accordance with UA levels (P = 0.804). Serum GGT is a widely used marker for fatty liver disease and alcohol consumption and is associated with an increased risk of CAD [29]. Huang et al. reported that GGT was associated with the risk of ACS in relatively young patients [8]. In the current study, GGT was associated with CAD in young patients according to the Mann–Whitney U test, but not significantly associated with CAD by univariate analysis. Therefore, we have shown that there is no relationship between serum GGT and CAD in a young population. In our study, the levels of aspartate aminotransferase and alanine aminotransferase were similar in both groups, and we think that this may be dependent on the absence of cardiomyocyte necrosis in SAP. As a result, contrary to the information set out so far, inflammation may not play an important role in the pathogenesis of atherosclerosis in stable patients. 

This study had some limitations. First, we used only single laboratory measurements of parameters for our patients, and repeated, longitudinal laboratory measures are more informative. Second, the study is based on retrospective data. Third, data on drug use were not complete. Fourth, modernization associated with a sedentary but stressful lifestyle and drug abuse are suggested as additional nontraditional risk factors for CAD, especially in young patients; we did not evaluate these parameters in our study. Fifth, current markers that have recently been mentioned in the development of atherosclerosis, such as fibrinogen, sialic acid, serum protein amyloid a, and C-reactive protein, among others, could not be evaluated in our study.

In conclusion, contrary to studies done in the elderly, well-known traditional (hypertension and diabetes) and nontraditional risk factors (except low eGFR) could not exactly predict the presence of CAD in a young population with SAP. Only advanced age, smoking status, and lipid profile were significantly associated with the presence of significant CAD in young patients with SAP. Future studies are needed to explain the presence of CAD in young patients, its pathological mechanisms, and its relationship with traditional and nontraditional risk factors.
